# Changes in Intimacy and Sexuality During the COVID-19 Pandemic: A Qualitative Analysis of Data from a Survey on Partnered Individuals in Eight European Countries

**DOI:** 10.1007/s12119-022-10035-1

**Published:** 2022-10-27

**Authors:** Charlotta Löfgren, Eva Elmerstig, Johanna Schröder, Marie Chollier, Jasmina Mehulić, Hanneke de Graaf, Katerina Klapilova, Inês Tavares, Peer Briken, Özlem Köse, Pedro Nobre, Aleksandar Štulhofer

**Affiliations:** 1 Faculty of Health and Society, Malmö University, Malmö, Sweden; 2 Centre for Sexology and Sexuality Studies, Malmö University, Malmö, Sweden; 3 Institute for Sex Research, Sexual Medicine, and Forensic Psychiatry, University Medical Centre Hamburg-Eppendorf, Hamburg, Germany; 4 Department of Psychology, Medical School Hamburg, Hamburg, Germany; 5 APHM, Marseilles University Hospital, Marseille, France; 6 Faculty of Humanities and Social Sciences, University of Zagreb, Zagreb, Croatia; 7 Rutgers, Dutch Centre of Expertise on Sexuality, Utrecht, The Netherlands; 8 National Institute of Mental Health, Klecany, Czech Republic; 9 Faculty of Psychology and Education Sciences, Center for Psychology at the University of Porto, University of Porto, Porto, Portugal; 10 Independent Relationship and Sex Researcher in Private Practice, Montreal, QC Canada

**Keywords:** COVID-19, Reflexive thematic analysis, Adults, Relationship satisfaction, Sexual health, Intimacy

## Abstract

This qualitative study explores experiences of intimacy and sexuality during the first phase of the COVID-19 pandemic of 3357 participants from Croatia, the Czech Republic, the Netherlands, France, Germany, Portugal, Sweden, and Turkey. Data were collected through open-ended questions within a survey on sexuality during the pandemic. Based on a reflexive thematic analysis three themes occurred. The first theme *No change* was described by 41% when summarizing their sex life during the pandemic. The second theme *Positive change* was experienced by 25%, and the third theme *Negative change* by 21%. An overarching theme then emerged as: “Still the same trajectories, but the pandemic could be a catalyst for improved or worsened sex- life.” For those intimate partnered individuals who already had problems with intimacy and sexuality before the COVID-19 pandemic it seemed to remain the same or deteriorated throughout the pandemic. For those with positive experiences of intimacy and sexuality before the COVID-19 pandemic it remained the same or improved throughout the pandemic. These findings are relevant for researchers and clinicians in developing preventive and supportive interventions in the context of crisis and social isolation.

## Introduction

Since the World Health Organization declared COVID-19 a pandemic on March 11, 2020 (WHO, 2020), different levels of societal restrictions (e.g., lockdowns) have been introduced in Europe, as well as in the rest of the world. These restrictions changed living conditions for non-partnered as well as partnered individuals (e.g., Arafat et al., 2020; Banerjee & Rao, 2020; Feng et al., 2021; Luetke et al., 2020). The latter either remained apart (i.e., in different cities or countries due to travel restrictions) or were forced to – for better or worse – stay at home together (Gunther-Bel et al., 2020). There is a growing body of knowledge on how the COVID-19 pandemic has affected and still affects sexuality, sexual health, sexual behavior and sex life quality (e.g., Mitchell et al., 2022; Panzeri et al., 2020; Štulhofer et al., 2022; Vigl et al., 2021; Wignall et al., 2021). But, qualitative data focusing solely on cohabiting partnered individuals’descriptions of their sex-life during the pandemic are currently scarce. This article aims to contribute to the understanding of partnered individuals’ sex lives during the initial phase of the COVID-19 pandemic. While most previous studies used quantitative methods to address this issue, our study used a qualitative approach to provide more knowledge about and subjective meanings related to the phenomenon.

### Sexuality and Sexual Health in Partnered Individuals During the COVID-19 Pandemic

The importance of sexual relations, sexual health, and sexuality as significant determinants of relationships and general well-being are well documented (e.g., Laumann et al., 2006; WAS, 2010/2021; WHO, 2006/2021). Social interaction, intimacy, and fulfilling close relationships have been shown to boost well-being and serve as critical coping factors during crises (Banerjee & Rao, 2020). However, due to the risk of being infected by COVID-19, current research shows fear of intimacy and sexuality (ibid.). This was also observed by Delcea et al., (2020) who found a decrease in sexual activity in individuals during March and April 2020 when they conducted a meta-analysis on quantitative studies of sexual health during the pandemic in the US, China, Turkey, Italy, UK, and Ireland.

A Chinese survey showed that sexual behavior factors and family functions were important determinants of partner intimacy, referring to romantic relationships, and addressed its importance for sexual health (Feng et al., 2021). Another Chinese cross-sectional online survey concluded that health strategies and guidelines are needed to safeguard sexual and reproductive health during the COVID-19 pandemic (Li et al., 2020). Likewise, several European studies call for action and international guidelines for sexual health (e.g., Barbee et al., 2020; Hall et al., 2020; Pennanen-Iire, 2021).

Nevertheless, the knowledge regarding if and how the pandemic creates several specific challenges for emotional intimacy and sexuality among partnered individuals is limited. In a cross-cultural study carried out in three south-east Asian countries, Arafat et al., (2020) showed that 45% of the participants reported that isolation due to COVID-19 had an impact on their sex life. During the lockdown, 72.5% reported having sex 1 to 5 times a week, an increase of 3.3% in sexual activity compared to the time before the pandemic. Furthermore, 50% reported having positive changes in their emotional bonds (ibid.). A Turkish study focusing on the effect of the COVID-19 pandemic on partnered females’ sexual behavior showed that sexual desire and frequency of intercourse significantly increased during the COVID-19 pandemic, whereas perceptions of the quality of sexual life at the same time significantly decreased (Yuksel & Ozgor, 2020). A brittish study investigad the extent to which the lockdown during the first phase of the COVID-19 pandemic affected steady relationships and sex life quality (Michell et al., 2022). They found that one fifth experienced no change regarding their sex life quality, and more than half reported no change in their relationship quality.

Luetke et al., (2020) showed in a US study that 35% reported some degree of conflict with their romantic partners due to the spread of COVID-19 and its related restrictions. According to Balzarini et al., (2021), people who reported greater COVID-19-related stressors also reported feeling less satisfied with and less committed to their relationships, and reported more conflicts in their relationships. Štulhofer et al., (2022) argue in a quantitative study of seven European Union countries and Turkey that the COVID-19 pandemic may be challenging because there is no reliable narrative and overview about the pandemic’s dynamics. Variations were shown in this cross-cultural study on changes in sexual interest and distress related to discrepant sexual interest during the COVID-19 pandemic within cohabiting partnered individuals (Štulhofer et al., 2022). The results showed that more than half (53%) of the participants reported no change in their sexual interest, 28.5% an increase, and 18.5% a decrease (ibid.). However, an Italian study investigating cohabiting partnered individuals showed that most couples did not perceive any differences in their sexuality related to the COVID-19 lockdown in April–May 2020 (Panzeri et al., 2020). Those who did report decrease in pleasure, satisfaction, desire, and arousal, stated worries, lack of privacy and stress as primary reasons.

These changes observed in different studies have been associated with a range of contextual, individual, and interpersonal/dyadic factors. Carvalho and Pascoal (2020) stated that the ability to adapt to contextual changes such as the COVID-19 pandemic is a key feature characterizing relationships with higher levels of perceived satisfaction and marital quality. Uncertainty and misinformation about the routes of transmission for COVID-19 and, consequently, fear of contagion might lead to avoidance and/or difficulties related to intimacy and sexuality (Banerjee & Rao, 2020; Marano et al., 2021). As some individuals may use sex to cope with negative mood or stress (e.g., Bodenmann et al., 2007; Carvalho & Pascoal, 2020; Gillespie et al., 2020), the lockdown and social isolation may, according to Carvalho and Pascoal (2020, p. 1214) lead to “maladjusted sexual behavior,” or to new or increased sexual behavior (e.g., increased porn use) that may alter the couple’s balance (Mestre-Bach, 2020; Zattoni et al., 2020). Lehmiller et al., (2020) also addressed the importance of creatively adapting sexual life to the pandemic situation. Their online study in the United States showed that nearly half of the sample reported a decline in their sex life, and that one in five reported expanding their sexual repertoire, for example to include sexting, new sexual positions, and sexual fantasy sharing (ibid.). Furthermore, the chance of an improved sex life was three times higher among those who had tried something new than among those who had not (Lehmiller et al., 2020). In line with this, Lopes et al., (2020) suggested that sexual counselling during the pandemic, when needed, can assist in adopting new sexual routines and reinventing sexual intimacy.

So far, sexuality-related research since the COVID-19 pandemic outbreak has covered a wide spectrum of foremost quantitative knowledge of experiences and behaviors, and on how people’s sex lives are affected by the ongoing situation. Only a few offer knowledge and subjective interpretations of and meanings attached to changes in sex life (e.g. Rubinsky et al., 2022), which are essential for a fuller understanding of quantitative findings (Panzeri et al., 2020). Neither does previous research focus on cohabiting partnered individuals’ sexual experiences.

This study aims at filling this gap by providing a qualitative analysis of open-ended answers within a survey on partnered individuals in eight European countries, where they describe their sex life during the COVID-19 pandemic (Štulhofer et al., 2022).

### Aim and Research Questions

This current paper aims to expand the understanding of how partnered individuals experienced their sex life during the first phase of the COVID-19 pandemic. In 2020, a large-scale online study explored how partnered individuals experienced intimacy and sexuality during the first 2–4 months of the COVID-19 pandemic in Croatia, the Czech Republic, the Netherlands, France, Germany, Portugal, Sweden, and Turkey (Štulhofer et al., 2022). The final item in the survey was an open-ended question: “Finally, we would like to ask you to write one (or two) sentences that summarize your sex life during these times of pandemic”. In this analysis, we utilized the qualitative data from the answers, following two research questions:

1. How do partnered individuals describe their experiences of emotional intimacy and sexuality in the context of the COVID-19 pandemic?
2. Did the pandemic cause any perceived changes in emotional intimacy and sexuality, and if so, how are these changes explained?

## Method

### Choice of Method

The research team opted for a qualitative approach, using Braun and Clarke’s (2019) reflexive thematic analysis (TA), to answer the research questions. The researchers’ subjectivity is thereby understood as a resource, and the process aims to be creative, reflexive, and subjective (ibid., p. 591):

Qualitative research is about meaning and meaning-making, and viewing these as always context-bound, positioned and situated, and qualitative data analysis is about telling ‘stories’, about interpreting, and creating, not discovering and finding the ‘truth’ that is either ‘out there’ and findable from, or buried deep within, the data.

Our data are unique, due to the large number of statements from informants’ that have conveyed their experiences. However, the data differ from individual interview data, while it lacks opportunities to ask follow-up questions and contains of short statements. Therefore, we will describe our subjectivity and reflexivity as researchers in this organic process of coding when generating themes, just as Braun and Clarke (2019) suggest. Themes are described as “patterns of shared meaning underpinned or united by a core concept” (ibid., p. 5). If more than one researcher is involved, Braun and Clarke describe the analytic process as collaborative and reflexive; “designed to develop a richer more nuanced reading of the data, rather than seeking a consensus on meaning” (ibid., p. 7). In this article, two experienced sex researchers acted as research leaders within the European research team—one with a medical and one with a social science background. The research leaders continuously analysed and discussed the process with the whole research group, that consisted of sex researchers, from the eight countries included, with different scientific backgrounds, e.g., medical, sociological, psychological. Our joint interdisciplinary analysis made the process trustworthy.

### Participants and Recruitment

Participants were recruited as part of a larger online survey with eight European countries, housed by one institution/country. Inclusion criteria were cohabiting partnered individuals over 18 years. The participants were recruited via national news websites, and online social networks. The study was advertised as focusing on possible impact of the pandemic on intimacy and sexuality in longterm relationships. All participants were asked the same questions in eight different languages. As described more in detail in Štulhofer et al., (2022), 4,813 respondents (*M*_age_ = 38.5, *SD* = 10.74) from eight countries Croatia (*n* = 492), the Czech Republic (*n* = 537), France, (*n* = 358) Germany (*n* = 529), the Netherlands (*n* = 926), Portugal (*n* = 476), Sweden (*n* = 356), and Turkey (*n* = 1139), completed an online survey between May and July 2020. Median time to complete the questionnaire was below nine minutes, and there were no compensation offered.

A total of 3,357 participants completed the following open-ended question at the end of the online questionnaire: “Finally, we would like to ask you to write one (or two) sentences that summarize your sex life during these times of pandemic”. As shown in Table 1, participants who completed the open-ended questions were from Croatia (*n* = 323), the Czech Republic (*n* = 402), the Netherlands (*n* = 714), France (*n* = 264), Germany (*n* = 365), Portugal (*n* = 281), Sweden (*n* = 270), and Turkey (*n* = 738). The majority of respondents self-identified as women (*n* = 2448, 72.9%). Mean age in the sample was 39.2 years (*SD* = 11.05; range 18–80). Nine participants had another gender identity than women or men, and 11 reported being in another type of partnership than same-sex or opposite-sex relationships. A total of 55.4% had children in their household.

**Table 1:** Sociodemographic characteristics of the sample (N = 4813) and the qualitative sub-sample (N = 3357)

	Survey sample n (%)^a^	Participants who responded to the open-ended question n (%)^a^	Item response rate
Country			
Croatia	492	323	65.6%
The Czech Republic	537	402	74.9%
France	358	264	73.7%
Germany	529	365	69.0%
The Netherlands	926	714	77.1%
Portugal	476	281	59.0%
Sweden	356	270	75.8%
Turkey	1139	738	64.8%
Gender			
Women	3501 (72.7%)	2448 (72.9%)	69.9%
Men	1300 (27.0%)	898 (26.8%)	69.1%
Other	12 (0.2%)	11 (0.3%)	91.7%
Children in the household			
Yes	2657 (55.2%)	1858 (55.4%)	69.9%
No	2155 (44.8%)	1499 (44.7%)	69.6%
Type of partnership			
Opposite sex	4553 (96.9%)	3283 (97.8%)	72.1%
Same sex	146 (3.1%)	63 (1.9%)	43.2%
Being able to have some privacy in the household during the pandemic			
Yes	3166 (71.6%)	2404 (71.6%)	75.9%
No	1255 (28.4%)	950 (28.3%)	75.7%
	*M (SD)*	*M (SD)*	
Age	38.5 (10.7)	39.2 (11.05)	
Years of completed formal education	15.9 (3.7)	15.9 (3.6)	
Years of living together with the partner	10.6 (9.5)	10.9 (9.8)	

^a^Percentages do not always add up to 100 due to rounding up

### Translation Process

The survey in the target languages was administered online using GDPR (General Data Protection Regulation)-compliant software. Since answers to the open-ended question were completed in eight different languages, the initial dataset included multilingual answers. To enable the analysis, all answers were first translated to English within each countries’ research teams. Each set was then checked by a second bilingual researcher and/or professional for most of the participating countries. Specialty in the field was deemed important to ensure not only semantic but also conceptual equivalence; this process ensured a rigorous translation and transliteration process for a reliable analysis (Regmi et al., 2010).

### Data Analysis and Coding Phases

The large amount of data collected led to a dual process between the two research leaders and the whole research group, consisting of both deductive and inductive researcher-based coding (Braun & Clarke, 2019; Fereday & Muir-Cochrane, 2006; Firmin et al., 2017). We used the software program MAXQDA (Kuckart & Rädiker, 2019), and then explored the data and built a coding tree within the program. The coding tree was developed closely based on the empirical data, in an active and generative process, containing thoughtfulness and reflection according to reflexive TA (Braun & Clarke, 2019).

First, the two researchers reviewed the translated data material, mapped it, and searched for keywords. Most of the participants mentioned change (or the absence thereof) in the summary of their sex life during the COVID-19 pandemic. Thus, change became the key concept in the coding. Next, we coded and themed the responses (meaning units) into the following themes: No change, Positive change, Negative change, and Ambiguous or unclear statements. The latter theme was omitted from the final analysis, as it did not connect to the research questions and/or was not understandable or not possible to analyze further (e.g., only an emoji or symbol). The size of this theme was 417 statements (12.4%).

In the third phase, the themes were linked together with codes, such as within No change: “As good as before,” and “As bad as before,” within Negative change: “No privacy, too close,” “Stress, sickness, quarrel, divorce,” and “Lack of other sex partners inside or outside the relationship.” Lastly, within Positive change: “More intimacy, closeness,” “More time, less stress,” and “New ways of having sex.” Throughout the analysis, the two researchers looked for patterns, tendencies, and underlying meanings of change and explanations for change. Thus, the themes generated an over-arching theme: “Still the same trajectories, but the pandemic could be a catalyst for improved or worsened sex-life”. (Table 2).

**Table 2:** The Overarching Theme, Themes, Codes and Meaning units identified from the analysis of the qualitative data

Overarching theme	Still the same trajectories, but the pandemic could be a catalyst for improved or worsened sex-life							
Themes	*No change* – “Pandemic did not affect our sex life”		*Positive change* – “We are more satisfied”			*Negative change* – “Lack of space for both home-office and sex”		
Codes	As good as before	As bad as before	More intimacy, more closeness	More time, less stress	New ways of having sex	No privacy, too close	Stress, sickness, quarrel, divorce	Lack of other sex partners
Examples of meaning units	The pandemic does not affect my sex life, it is as great and frequent as before	Same as before. Because, parallel with COVID-19, our relationship is going through a crisis for another reason, there is less sex than before, like a year ago	The sexuality is very tender, more closeness and caressing	More time spent together and less tension regarding social situations and work have made it so that we are enjoying each other more often and more freely	Trying out new caresses, that leads us to more intense emotional feelings. We love to drive ourselves to orgasm again and again	Since being together too often has reduced our interest for each other, we have sex life with less emotional intensity than before	The intensity of the interaction makes small dramas become big	The constant presence of children, tedious routine, no sauna, no lover, no evening that makes you want

Braun and Clarke (2019, p. 593) conceptualize themes as “patterns of shared meaning underpinned or united by a core concept.” The interpretations of our findings were discussed among the co-authors throughout the process, which meet the needs of the analysis’ trustworthiness (c.f. Nowell et al., 2017).

## Results

Less than half of all participants (N = 1,390; 41.4%) fell under the theme No change when summarizing their sex life during the first phase of the COVID-19 pandemic. The other half consisted of 830 (24.7%) who fell under the theme Positive change and 720 (21.4%) who stated Negative change. Finally, 417 (12.4%) fell under the theme Ambiguous or unclear in relation to change and was deducted from the final analysis. The pattern was similar across the participating countries.

### No Change—"Pandemic Did Not Affect our Sex Life."

The participants who fell under No *change* (*n* = 1,390, 41.4%) described their sex life in positive or negative ways. Therefore, the statements of *No change* were divided into “As good as before” and “As bad as before.”

#### As Good as Before

Quite a few of those who stated *No change* described their sex life in positive ways in relation to how they also described their relationship before the pandemic, for example “As perfect as always." Some of the participants referred to frequencies in their sex life and assessed whether it had increased or decreased. If not, it was seen as a satisfactory and stable sex life. A participant from the Czech Republic wrote:

Nothing has changed. We have sex at least 3 times a week. Both before the pandemic and now.

[THE CZECH REPUBLIC, woman, age 29, opposite-sex relationship]

Others described their sex life in positive terms, without mentioning frequencies. A Swedish participant stated:

Satisfactory and no difference from before the pandemic.

[SWEDEN, woman, age 46, opposite-sex relationship]

The ability to adapt to changes seemed to increase the options to maintain what is perceived as a good sex-life during crisis. Several participants described the importance of flexibility and adjustment to new situations as a key factor of a positive relationship and satisfactory sex-life. A participant from Portugal wrote:

My husband and I are very active, we have always taken time for our intimacy, the pandemic has not changed anything. He looks for it more but the two of us have a lot of desire for each other. We had to adapt to a lot in the pandemic, but we are used to dealing with a lot when we have 5 children, however we try to make sure that our couple time is not affected.

[PORTUGAL, woman, age 37, opposite-sex relationship]

Furthermore, emotionally stable, and loving relationships were described as a well-grounded foundation when the participants within this theme confronted the crisis. The participants who were satisfied with their sex life and their relationships pointed out that the pandemic could not change that. A participant from Croatia stated:

Nothing can endanger my love for my partner, not even a tiny virus.

[CROATIA, man, age 56, opposite-sex relationship]

#### As Bad as Before

The opposite from those who stated “As good as before” also became clear and obvious among the participants who stated *No change*. “It was bad, and it is still bad” concluded the statements from these participants who summarized their sex life in a consistently negative way. Previous sexual problems, sexual dysfunctions, relational problems, and emotional distance were stated as backgrounds to their reports. Several participants described sexless relationships both before and during the pandemic. A French participant stated:

Before, during, after the pandemic, no sex life with my partner.

[FRANCE, woman, age 45, opposite-sex relationship]

Because of problems in their relationship in general, several had a non-existing sex life or rare sexual interactions, not connected to the pandemic. A Turkish participant said:

Once a month is more than enough with my partner, if possible. My problem is not the pandemic, it's my partner.

[TURKEY, woman, age 48, opposite-sex relationship]

More than several participants stated that their sex-life had not changed due to the pandemic. Instead, it was influenced by ordinary caring duties and responsibilities connected to relationships and family life, such as having children, struggling with teenagers, having puppies, elderly parents, and so on. “Children” was one of the most frequent words in the participants’ answers and referred to tiredness, a lack of privacy and time for the sexual relationship. In line with this, one woman (age 26, opposite-sex relationship) from Germany described the current situation as: "significantly less sex-life through existing early pregnancy." A participant from Czech Republic stated:

Our sex life is affected by two toddlers at home. That has not changed with the arrival of a pandemic.

[CZECH REPUBLIC, woman, age 40, opposite-sex relationship]

### Positive Change—"We are More Satisfied"

Participants (*n* = 830, 24.7%) who stated *Positive change* during the pandemic compared to their sex life before the pandemic, described it in different ways: “More intimacy, closeness,” “More time, less stress”, and “New ways of having sex.”

#### More Intimacy, More Closeness

For participants who described their sex life during the pandemic as rewarding, healing, gratifying, and spontaneous, these experiences were connected to descriptions of closeness, intimacy, and affection, as well as to a sense of living in a loving relationship. A participant from France described this:

Love at all levels. We have the impression of being centred on the essential, connected to each other. Beyond the problems, the disease, the vagaries of life, there is something more essential: love.

[FRANCE, woman, age 46, opposite-sex relationship]

Because of the COVID-19 situation, several participants stated that they became aware of the importance of health, love, and relationship. A Turkish participant stated:

At first it was a bit of anxiety, but then it recovered as it got used to the situation. A little better than before the pandemic because we realized that the most important thing in life is health and love.

[TURKEY, woman, age 35, opposite-sex relationship]

A German woman (age 62, same-sex relationship) described their sex life as: “Strengthening, building, relieving,”, just as a French woman (age 52, opposite-sex relationship) stated: “Fulfilling and renewed. Full of novelty.” Furthermore, many participants stated that they valued their partner more and grasped the moment of expressing intimacy and closeness within their relationship during the pandemic. A participant from the Czech Republic stated how their relationship had improved during the pandemic:

More intimacy, more touches, noticeable easing into lovemaking at any time during the day without bothering what and when to plan. Better atonement and connection to a partner, removing blocks and opening taboo topics of our sex-life.

[CZECH REPUBLIC, woman, age 36, opposite-sex relationship]

In line with this, many participants stated that they were paying more attention to each other’s health and well-being. A participant from Germany stated:

We are still fundamentally as close to each other as we were before the pandemic, but we are paying even more attention to each other and have become more empathetic/open to each other's concerns.

[GERMANY, woman, age 24, opposite-sex relationship]

#### More Time, Less Stress

Several participants described the importance of having more time and being under less job-related stress, which enabled a more gratifying sex life. Furthermore, several couples described how, before the pandemic, they were often exhausted after work and family care. Lockdown and remote work made it possible to have sex at previously unusual times, for example during daytime, when they were more alert and had more energy. A participant from the Czech Republic stated:

We can be sexually active also outside evening hours, which is good for our sex- life.

[CZECH REPUBLIC, woman, age 29, opposite-sex relationship]

Less fatigue and more time for the couple was oft-repeated statement, regardless of the country of residence. That also meant that partnered individuals had more time to sleep and felt less stressed. Another participant from the Czech Republic described using sex as a coping mechanism while under work-related stress:

We have more time, we sleep longer. We are more relaxed, but mentally tense. We have more work-related stress — sex helps among other things to release the stress.

[CZECH REPUBLIC, man, age 31, opposite-sex relationship]

Moreover, several participants described that they used this access to more free time for sexual activities, not only for sleep. One participant from Germany stated:

We use more free time for sex and sexual activities.

[GERMANY, woman, age 23, opposite-sex relationship]

Making the best of a tough period was described by several participants across countries. More time during daytime also helped some couples remember the first phase of their relationship and sex-life. One participant from Portugal wrote:

Remember the wild times of passion! Innovate and take advantage of the time we didn't have before the pandemic (try to see the positive).

[PORTUGAL, woman, age 36, opposite-sex relationship]

Even though several participants described a family situation with children and teenagers living together during the pandemic as taxing (see Negative Change), there were also those who appreciated a closer family-life with more time together. This can also be a suitable time to become pregnant, for those who wish to have children without the usual everyday stress. A woman (age 30, opposite-sex relationship) from the Czech Republic stated that “during the pandemic, I finally got pregnant after 3 years of trying.” In line with this, a participant from Germany described how the COVID-19 pandemic led to a desire to have children, which in turn led to more sex:

A desire to have children has definitely arisen which was not there before. So now we explicitly pay attention to having sex on the fertile days, which we left to chance before.

[GERMANY, woman, age 31, opposite-sex relationship]

#### New Ways of Having Sex

Some participants mentioned experiences of new ways of having sex as one positive change during the COVID-19 pandemic. Internet or virtual sex was mentioned as, for example, looking at erotic or pornographic films or pictures. One French woman (age 45, same-sex relationship) stated the word "virtual" while describing their sex life. Some describe the option to have cybersex either through web camera or via “sexting” (text messages or images with a sexual content). This was seen as a positive option for couples who could not be together physically during the pandemic, for example when one went away on a work-trip or had to take care of their old parents, or lived in a poly-relationship. A Swedish participant states:

Sex through FaceTime with my partner has become an opportunity.

[SWEDEN, woman, age 53, opposite-sex relationship]

Other new ways of having sex that the participants mentioned were role-play and using sex-toys. A participant from the Netherlands described the positive ways that their sex- life improved during the pandemic:

Fantastic. Trying out several new things as we spend more time together. All kinds of positions, toys and role-play.

[THE NETHERLANDS, man, age 23, opposite-sex relationship]

Furthermore, new ways of having sex seemed to connect to a relationship that already was stable and included trust and loving aspects. The COVID-19 pandemic was then seen as an option for discovery of sexual variations. A participant from Germany stated:

It's fun, and we discover new things. there is a lot of trust between the two of us, and this is the basis for new discoveries.

[GERMANY, man, age 71, opposite-sex relationship]

New ways of having sex were also associated with opportunities to have sex at other times than usual, for example during the day instead of in the evening. That made some of the couples more open to sexual variations. A Croatian man (age 22, opposite-sex relationship) said: "We have more time to experiment." In line with this, a Swedish participant stated:

We have been more innovative. Sex at lunch, and so on.

[SWEDEN, woman, age 40, opposite-sex relationship]

### Negative Change—"Lack of Space for Both Home-Office and Sex”

Around one fifth of all participants (720, 21.4%) fell under *Negative change* during the COVID-19 pandemic. These changes were described in statements connected to the following themes: “No privacy, too close,” “Stress, sickness, quarrel, divorce,” and “Lack of other sex partners inside or outside the relationship.”

#### No Privacy, too Close

Given the fact that a lot of couples experienced a new situation with different levels of quarantine due to the pandemic, the consequences of home or remote work and home-schooling also influenced the sex-life of several individuals. Some described having a lack of privacy as distressing, especially for those who otherwise appreciated the space when going away from home during the day, as one woman (age 22, opposite-sex relationship) form the Czech Republic explained: “Disturbing, I miss the privacy and intimacy because we cannot go anywhere.” Furthermore, this was described as if the attraction and the positive tension with their partner had disappeared. Therefore, their sex life deteriorated, as one French woman (age 37, opposite-sex relationship) states: “Living together 24/7 is a turn off.” One participant from Germany stated:

At first, I thought we would have more time for sex now. But now we both work from home and can hardly separate work and private life (even physically). We also spend about 22-23 of 24 hours together. Now we have sex less often than usual.

[GERMANY, woman, age 33, opposite-sex relationship]

Working at home also influenced couples’ possibilities for privacy during the COVID-19 pandemic. For example, tasks such as digital teaching and digital meetings that require a web camera put an extra dimension on losing a sense of privacy, blurring boundaries between professional and private life and spaces. A participant from Portugal described how this influenced the couple’s sex-life:

I am overwhelmed. I have telework, classes from home to manage, and the management of the home. I feel embarrassed that I don't have privacy and space for romance; sex is the last thing I think about.

[PORTUGAL, woman, age 44, opposite-sex relationship]

#### Stress, Sickness, Quarrel, Divorce

A number of participants were professionally overwhelmed by the COVID-19 pandemic, for example those who worked at hospitals, crisis centres or social welfare institutions: "I work on a crisis team. I am more overworked and tired than before the crisis and therefore I have less desire for sex,” one woman (age 29, opposite-sex relationship) from the Netherlands described. Experiencing an extraordinary weight at their jobs spilled over into their private life. This exceptional work situation occurring concurrently with private worries seemed to increase feelings of stress and fatigue. One participant from the Czech Republic described:

I am a medical student and I'm helping in a hospital. I am too tired for sex and generally I'm more exhausted, so it is unimaginable for me to put energy into sex. I miss motivation, I have an older partner who previously wanted sex less than I did, and now during the pandemic, it is vice versa.

[CZECH REPUBLIC, woman, age 21, opposite-sex relationship]

Furthermore, experiences of wearing a face-mask during the pandemic also influenced couples’ sex life in a negative way. According to a woman (age 43, opposite-sex relationship) from Germany, it led to tiredness: "Little change, frequency of event lower because of tiredness caused by constantly wearing a mask." A Czech Republican woman (age 37, same-sex relationship) mentioned that the face- mask influenced the individual’s way of showing sexual emotions, with kisses excluded. Not least, several mentioned the risk of being infected by COVID-19 via their partner. A participant from the Netherlands described the consequences of this for their sex-life:

My partner doesn't want to have sex now because he finds it scary that I come into contact with other people for my work. I don't mind because I don't have a high libido. That's fine.

[THE NETHERLANDS, woman, age 45, opposite-sex relationship]

Stress and pressure can lead to irritation, quarreling, and sometimes to separations and divorces. Sometimes this was described as consequences of the pandemic, as one Turkish woman (age 38, opposite-sex relationship) described: "Either take him/her (partner) from home or take me out of home." Nevertheless, it can be difficult to know the origin for quarrel and problems within a relationship. Relational problems might not be related directly to the COVID-19 pandemic, but to other problems such as cramped housing or financial issues. The pandemic could then make this situation even worse. "The intensity of the interaction makes small dramas become big,", as one Portuguese man (age 40, opposite-sex relationship) put it. Additionally, some participants have lost their jobs during the pandemic, which influenced them emotionally as well as financially. Therefore, it also impacted relationships and sex-lives. A Turkish participant described their situation:

Pandemic has affected the relationship between me and my partners from my perspective, not because of the fear of catching the virus but because of some reasons such as not being able to work, mistrust and examining my emotions thoroughly. While my partners´ respect and love increased towards me, I started to lose not my respect but my love and, because of my concern for the future, trust for him/her.

[TURKEY, woman, age 40, living with more than one person]

In other instances, the COVID-19 pandemic has made individuals within a relationship realize how they want to live their lives, what is important, and also how they want to live in the future. Some participants stated that they had decided to separate during the pandemic. A French participant described their situation:

The pandemic made me realize my sexual anorexia and the huge gap that there was with my partner. While I was killing myself at work, he was killing himself to satisfy his sexual and emotional needs that I no longer met. Today: I fully live my sexual anorexia and want to separate.

[FRANCE, woman, age 46, opposite-sex relationship]

Others described non-existing sex life, and situations when their partner had (sexual) contact with other persons outside their relationships without consent. The COVID-19 pandemic might then illuminate or worsen previous sexual challenges. A participant from Croatia stated:

Total zero. My husband has found his recreation in a woman on Facebook. And I can't say that it's the first time he has cheated on me, even virtually. The brain does its own thing when the hands are idle. *Adio mare*!

[CROATIA, woman, age 47, opposite-sex relationship]

#### Lack of Other Sex Partners

Some of the participant described a negative change within their sex-lives during the pandemic due to a lack of (sex-) partners inside or outside the relationship. It could be because of physical separation from their regular partner in another city, during for example a work-related trip, or one of the partners within a polyamorous relationship, or other partners while living in open relationships or partners in extramarital relationships or casual sex partners. As one Portuguese man (age 65, living with more than one person) described sex during the pandemic: "Poorer in the extra-marital part," in line with a man (age 55, opposite-sex relationship) from the Netherlands: "I have a mistress, but unfortunately I don't see her." A participant from Germany described the situation due to the COVID-19-restrictions:

Restricted because sex outside the relationship is currently not possible due to the risk of infection and contact restrictions. Also, the absence of third-party sex is missing. This leads to restrictions of sexual freedom.

[GERMANY, man, age 38, same-sex relationship]

Limited opportunities for flirting with other persons were also described by another man (age 53, opposite-sex relationship) from Germany as if "the erotic crackle in the partnership is missing.” One participant from the Netherlands wrote that they had agreed on an open relationship before the pandemic:

To date different people or try things sexually, as agreed within my open relationship with my steady partner. Because of corona this is not possible now, although I could only fulfil that need to a limited extent even before corona.

[THE NETHERLANDS, non-binary, age 23, relationship with a woman]

Some participants described more options for sex, but they also described less or no time with partners outside their household. A participant from Germany stated that they cancelled couple’s parties, but “continue[d] to have regular taboo-free and passionate sex.” Another participant from Germany described their current sex life during the pandemic:

For the first time, we enjoy having plenty of time for extensive BDSM sessions. However, we miss our partners, with whom we do not live together in the same household.

[GERMANY, woman, age 28, opposite-sex relationship]

Finally, a few of the participants stated the limited options of buying sex as a negative change due to the pandemic. A participant from the Netherlands described that closed “massage salons” led to more masturbation:

Little to no sex with my partner. All “massage” salons are closed, so a lot of self-work.

[THE NETHERLANDS, man, age 38, opposite-sex relationship]

### “Still the Same Trajectories, but the Pandemic Could be a Catalyst for Improved or Worsened Sex-Life”.

Finally, an over-arching theme occurred in the results: “Still the same trajectories, but the pandemic could be a catalyst for improved or worsened sex-life”. This overarching theme show that problems with intimacy and sexuality before the COVID-19 pandemic remained the same or deteriorated throughout the pandemic, which mirror a trajectory of stability or worsening. The overarching theme also show that positive experiences of intimacy and sexuality before the COVID-19 pandemic remain or improved throughout the pandemic, which explain the trajectory of stability or improving.

## Discussion

To sum up, our reflexive thematic analysis (Braun & Clarke, 2019) of the data provided by answers to the open-ended question from a multi-country survey on intimacy and sexuality showed similar patterns across all the participating countries. The overarching theme emerged as “Still the same trajectories, but the pandemic could be a catalyst for improved or worsened sex-life”. Almost half of the participants (*n* = 1,390) fell under the theme *No change* when summarizing their sex life before and during the first 2–4 months of the COVID-19 pandemic. Within this theme, about half described their sex-life in positive terms, “As good as before,” and the other half in negative terms, “As bad as before.” In the quantitative part of this project, 53% reported no change in their sexual interest during the pandemic (Štulhofer et al., 2022). One fourth of participants (*n* = 830) fell under the theme *Positive change* during the pandemic, for example: “More intimacy, and closeness,” “More time, less stress,” and “New ways of having sex.” In the quantitative part, 28.5% reported an increase in their sexual interest (ibid.). Finally, one fifth (*n* = 720) fell under *Negative change* in our qualitative part compared to the time before the COVID-19 pandemic, citing “No privacy, too close,” “Stress, sickness, quarrel, divorce,”, and “Lack of other sex partners.” Within the quantitative part of the project, 18.5% reported a decrease in their sexual interest (Štulhofer et al., 2022).

While our findings show that the COVID-19 pandemic seems to either maintain or worsen the tensions between partnered individuals within this theme, this could also influence sexual and mental health, as been shown in other current studies (e.g., Mitchell et al., 2022; Vigl et al., 2021). This is important to address, as recent research on sexual health during the COVID-19 pandemic indicates benefits of sexual activity on psychological, relational, and sexual health during the COVID-19 breakout (Mollaioli et al., 2021).

In our study, it became obvious that having children at home– especially babies and small children – is a stressful period for partnered individuals regardless the COVID-19 pandemic. This is also found in an American study, where having elementary aged children at home were associated with both reduced partnered bondings behaviors, as well as reduced partnered sexual behaviors (Hensel et al., 2020). During the COVID-19 pandemic our study also showed that it became demanding to have teenagers at home during daytime, home-schooling. These challenging family situations should be more investigated to support and unload partnered individuals during times such as the pandemic. For example, several Chinese studies point to the risk of home isolation for both domestic violence and divorce during the COVID-19 pandemic (e.g., Deese, 2020; Zhu et al., 2021).

Support centres via the Internet during the pandemic is one example of an important resource in order to prevent problems or offer counselling to partnered individuals and families (cf. Lopes et al., 2020). Luetke et al., (2020) also suggest professional support in assisting romantic partners to adapt and thrive in new and challenging conditions by for example promoting techniques to prevent and decrease stress and increase emotional support and intimacy.

For participants who described a rewarding long-term relationship, it seemed like their resources were enough to handle external crises such as the COVID-19 pandemic (cf. Banerjee & Rao, 2020). For some, more time together strengthened their relationship, and for others it was as good as before. Panzeri et al., (2020) found that most people do not perceive any difference in their sexuality related to the COVID-19. Nevertheless, knowledge about this group who is satisfied should be further investigated as this could be useful in developing support resources for couples who experience less satisfaction. Several of our participants who described positive experiences or positive changes also expressed the ability to adapt to new situations, which was likewise shown in a study by Carvalho and Pascoal (2020). For example, the willingness to try new ways of having sex is one interesting finding, which has also been shown in previous research during the pandemic (Lehmiller et al., 2020; Pereira et al., 2020).

### Further Research Questions

It is still impossible to fully review the consequences of the ongoing COVID-19 pandemic on intimacy and sexuality among partnered individuals. Therefore, there is a need for further qualitative and mixed-method studies to get a deeper understanding of how cohabiting and non-cohabiting partnered individuals describe and experience intimacy during a time of an external crisis such as the COVID-19 pandemic. It would be interesting to look further into partnered individuals’ coping strategies as well as adaptive abilities to better understand factors of importance for relationships. Not only risk factors should be identified, but also protective factors (cf. Vigl et al., 2021). This could be considered by policymakers in relation to decisions of future social restrictions (c.f. Wignall et al., 2021). Furthermore, it would be helpful in developing prevention programs, online and offline, and counselling models for those partnered individuals who already had or have developed negative and challenging sex-life trajectories during the COVID-19 pandemic or another external or internal crisis.

Gender, age and cultural factors are also important to further investigate in order to find patterns of similarities or differences in coping strategies among partnered individuals. Finally, further studies are needed to investigate the long-term effects of the COVID-19 pandemic on relationships and sexual health in order to identify clinical implications.

### Strengths and Limitations

The study has several limitations. First, it is not a typical qualitative one. We had a large amount of data in terms of participants but anchored in a single open-ended question within the questionnaire without opportunities to ask follow-up questions. Therefore, the data analysis is offering more of an overview than of qualitative traditional in-depth knowledge. Furthermore, difficulties in interpretation of short statements, consisting of one or two sentences, was also challenging. Some participants mentioned perceived reasons for their experiences, whereas some did not. Consequently, the statements could be described as snapshots of people’s experiences during the first phase of the pandemic. Additionally, our convenience sample is also a limitation. Who answered the open-ended-question?, and why? Our sample is clearly biased toward younger and more educated heterosexual women and men (see Table 1). Nevertheless, the patterns clearly showed that almost half of the participants did not describe any changes due to the pandemic, regardless of how they expressed experiences of intimacy and sexuality within their relationship.

Despite these limitations, there is a strength in the way we have collected data. This design allowed us to gather responses from a larger number of people than focus groups or individual interviews would have allowed for. In addition, the study consist of data from eight different countries, which make the results unique while most studies refer to one country. Furthermore, the participants’ comments offered exceptional insights into an extra ordinary global societal challenge during the COVID-19 pandemic.

## Conclusion

To conclude, we found that social distance and isolation with the “right” partner during the COVID-19 pandemic seemed to keep the relationship as good as before or even improved it in some ways. However, being in the same situation with “not the right” partner (at least in the circumstances of a pandemic) appeared to have a negative effect on the partnership, making sexual life either worse than before or at least as bad as before the pandemic. Overall, it seems that the pandemic, at least in its first phase, served as a catalyst for the important link between the quality of relationship and couple’s sex life.

The findings show several aspects of importance for researchers, clinicians, as well as social and medical health care professionals; for example, the importance of noticing that more time with each other makes it better only for those partnered individuals who already have positive sex-life trajectories. Not least, new research questions arise, for example concerning long-term effects of the COVID-19 pandemic on intimacy and sexuality among partnered individuals.
